# Digital technology innovation and agricultural industry chain resilience: implications for food security

**DOI:** 10.3389/fnut.2026.1915856

**Published:** 2026-08-26

**Authors:** Jie Chen, Jiayi Xu, Xintong Xiong, Yuqi Zhao, Huibin Yao

**Affiliations:** 1 School of Economics and Management, Changsha University of Science and Technology, Changsha, Hunan, China; 2 School of Cross-Border E-Commerce, Yango University, Fuzhou, Fujian, China; 3 School of Economics, Hunan University of Finance and Economics, Changsha, Hunan, China

**Keywords:** agricultural industry chain resilience, digital technology innovation, food security, heterogeneity, mediating effect

## Abstract

Food security is increasingly threatened by climate shocks, market volatility, and geopolitical disruptions. A resilient agricultural industry chain is critical to ensuring a stable food supply, yet empirical evidence on how firm-level digital technology innovation enhances such resilience remains scarce. Using micro-level data from Chinese agricultural firms over the period 2010–2025, which comprise 69 listed agricultural firms with 714 firm-year unbalanced panel observations, this study examines the impact of digital technology innovation on agricultural industry chain resilience. The results show that digital technology innovation significantly improves chain resilience, a finding that remains robust across multiple tests. Mediation analysis confirms that this effect operates through firm operational efficiency and market expectations. Heterogeneity analysis is conducted along two dimensions, namely regional heterogeneity and firm heterogeneity. Significant positive effects are found in eastern regions, urban agglomerations, and non-old-industrial bases from the regional perspective, and in non-state-owned, large-scale, and mature enterprises from the firm perspective. These findings provide empirical evidence for food-security-oriented policies that promote agricultural digitalization while addressing structural weak links in the food system.

## 1 Introduction

According to the Food and Agriculture Organization, the stability dimension of food security is defined as the long-term sustainability of food availability, access and utilization over time, such that these conditions are not interrupted by abrupt shocks including climatic extremes, market volatility or geopolitical conflicts^1^. On this basis, the resilience of agricultural supply chains, understood as the capacity to maintain essential operations in the face of exogenous disruptions, has become a core indicator directly relevant to food security, since its level fundamentally determines the stability of food provision (1, 2). In operational terms, resilience ensures that disruptions arising at any stage of the industry chain are contained before they escalate into systemic failures, whereas its absence permits localized shocks to propagate into widespread shortages and price volatility. Understanding the determinants of agricultural industry chain resilience is therefore a critical policy imperative for safeguarding food security in an increasingly volatile world. As a country with more than 1.4 billion people, China has long been confronted with challenges in its agricultural supply chain, including heavy reliance on foreign sources, conspicuous structural deficiencies and an imperfect operational system. When trade frictions, extreme weather events and public health emergencies converge as multiple external shocks, the risk of supply chain breakdown increases considerably, posing an immediate and severe threat to food security and to the stable supply of staple agricultural products (3). Nevertheless, it is worth noting that major Chinese agribusinesses, with the COFCO Group as a prime example, have risen to the front rank of global grain trading. These enterprises not only act as the backbone of China’s domestic food supply, but also function as indispensable nodes in the global food supply chain, and they are playing an increasingly significant role in stabilizing supply and demand patterns in international grain markets. In this context, strengthening the resilience of the agricultural supply chain has been unequivocally designated as a central priority in China’s national food security strategy and its agricultural modernization agenda (4). Unlike conventional approaches that confine their attention to agricultural production efficiency, China’s approach to supply chain resilience places greater emphasis on integrated governance, full-chain coordination and a bottom-line security mindset. This strategic reorientation, which moves from a reactive posture of coping with risk shocks toward a proactive pursuit of systemic stability, has directly strengthened the nexus between supply chain resilience and food security (5). At the same time, it demonstrates both the theoretical awareness and the practical means by which a large agricultural country can sustain its development trajectory in a complex and evolving international environment (6, 7).

**Figure 1:**
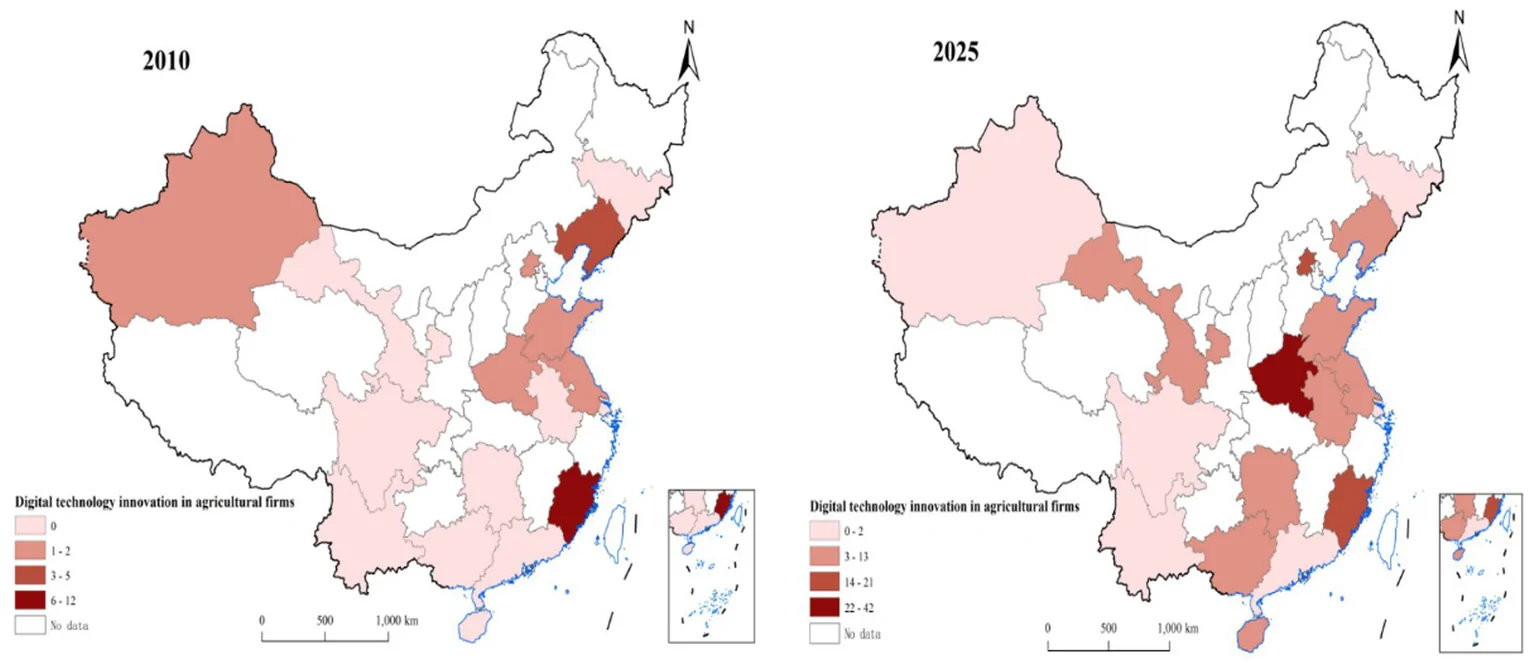
Regional distribution of agricultural digital innovation in 2010 and 2025.

Digital technology innovation, as a defining hallmark of the new round of technological revolution, has provided renewed momentum for the transformation and development of the agricultural sector. In practice, its application accelerates the deep convergence of digital factors with traditional production factors (8), enables precision decision-making in agriculture (9, 10), and facilitates the optimization of agricultural systems (11, 12). Against this backdrop, the regional landscape of digital technology innovation among Chinese agricultural enterprises is undergoing a notable transformation. As shown in Figure 1, based on technological innovation word frequency data from the CSMAR database, the regional distribution has shifted markedly: Fujian and Liaoning ranked among the top provinces in 2010, whereas by 2025, Henan had ascended to the leading position. This spatial reconfiguration thus reflects the changing developmental momentum across regions and the eastward-to-central shift in the center of gravity of agricultural digital transformation.

Yet, the relationship between digital technology innovation and agricultural industrial chain resilience is not unidirectional. On the one hand, digital technology innovation enhances synergistic collaboration among upstream and downstream actors along the chain (13, 14); on the other hand, it may also generate new competitive dynamics (15), thereby introducing novel risks that could directly threaten food security by disrupting supply continuity and price stability (16). This duality makes the enhancement of agricultural industrial chain resilience considerably more complex. These observations give rise to several critical questions: Does digital technology innovation exert a positive impact on the resilience of China’s agricultural industrial chain? What are its underlying mechanisms? Does heterogeneity exist? Answering these questions is essential for understanding whether and how digital technology innovation can be leveraged to safeguard food security.

The concept of resilience in academic circles has evolved from engineering resilience to ecological resilience and then to evolutionary resilience (17). Resilience originally emerged from ecology and engineering, where it emphasized a system’s capacity to maintain its structure and function in the face of disturbances. This concept was subsequently introduced into economics, where it gradually transitioned from a static definition of bouncing back to the original state to a dynamic interpretation of adaptation and adjustment (18, 19). It has since been further developed into a multidimensional framework encompassing the capabilities of resistance, recovery, reorganization, and path renewal (20, 21). This trajectory reveals a core consensus in contemporary resilience theory. A system’s resilience depends not only on its capacity to passively withstand shocks and return to normalcy but more fundamentally on its evolutionary capability to proactively adapt to environmental changes, achieve structural transformation, and foster path innovation. In the field of agricultural industrial chain resilience, scholars have drawn on the core tenets of resilience theory to forge a definitional consensus that has shifted from a narrow focus on resistance and recovery toward a broader paradigm centered on transformation. Some studies, motivated by the imperative of safeguarding food security and sustaining rural livelihoods, underscore the capacity of agricultural industrial chains to absorb shocks and achieve rapid recovery (22). Meanwhile, an expanding literature has broadened the conceptual framework by incorporating adaptation and transformation, maintaining that agricultural industrial chain resilience amounts to a multifaceted capacity encompassing the absorption of immediate shocks, the restoration of lost functionality, and the realization of sustained adaptive evolution through structural improvements and technological upgrading (23, 24). Synthesizing these perspectives, the existing literature generally conceives of agricultural industrial chain resilience as a multi-layered capability system that integrates resistance, recovery, reorganization and transformation (25, 26).

Regarding the factors shaping agricultural industry chain resilience, existing studies have identified the pivotal role of modern technology application. They argue that modern technologies, and digital technology in particular, are inherently replicable and transferable, and their spillover effects enable rapid integration into other industries, thereby accelerating the transformation of traditional sectors (27, 28), and exert profound effects on enhancing agricultural competitiveness, consolidating industrial stability, and reshaping resilience (29). However, constrained by insufficient digital infrastructure and limited digital resources, the integration of digital technology into agricultural industrial chains remains at a relatively low level. More critically, digital technology may act as an external shock to agricultural industrial chains and introduce new risks that could directly threaten food security by disrupting supply continuity and price stability (30). Evidently, the existing literature has yet to reach a consensus on the relationship between digital technology application and agricultural industrial chain resilience. In terms of research content, most studies focus on a single analytical dimension, whereas holistic research on agricultural industrial chain resilience remains scarce.

Building on this foundation, we synthesize insights from endogenous growth theory, induced technological change theory, and industrial structure evolution theory to construct a framework for analyzing how digital technology innovation affects agricultural industrial chain resilience and the mechanisms underlying this relationship. The sample comprises key enterprises positioned at the core of the global food supply chain, including prominent Chinese agribusinesses such as COFCO, and their strategic positioning renders the findings directly relevant to global food security governance. The study makes three marginal contributions.

To begin with, while existing food security research has predominantly relied on macro-level aggregate data and has thus left the micro-level behavioral foundations largely underexplored, we add micro-level empirical evidence and situate the findings within the broader food security discourse. Given that macro-level food system outcomes are an emergent property of the aggregated behavior of numerous micro-level firms and farmers, this study provides a theoretical justification for micro-level analysis from a digital innovation perspective, thereby helping to clarify the micro-foundations of stable food provisioning. Furthermore, although prior studies have acknowledged the potential importance of digitalization for food systems, they have remained largely at the level of macro-theoretical exposition without empirically identifying the specific transmission channels through which digital innovation operates, we identify a dual-channel transmission mechanism and elucidate its implications for food security. While previous work has remained largely at the level of macro-theoretical exposition, this study employs micro-level data to demonstrate that digital technology innovation bolsters industrial chain resilience through two distinct pathways, operational efficiency and market expectations. By doing so, it addresses the conditions under which the stability dimension of food security can be achieved. Finally, whereas most existing studies have treated the effects of digital innovation as homogeneous across contexts and have thus offered limited policy differentiation, we conduct a multidimensional heterogeneity analysis designed to inform differentiated food security policies. The systematic examination of heterogeneous effects encompasses both regional and firm-level dimensions, thereby providing a basis for targeted strategies that respond to the diverse requirements of global food security governance.

## 2 Theoretical analysis and research hypotheses

### 2.1 Direct effect of digital technology innovation on agricultural industry chain resilience

The reason why digital technology innovation in agricultural firms can influence agricultural industrial chain resilience lies fundamentally in the deep-seated fit between its technological attributes and the operational logic of agricultural industrial chains.

First, digital technology innovation is characterized by pronounced pervasiveness and ubiquitous connectivity. Agricultural industrial chains are complex systems spanning production, processing, distribution and sales, with stages highly interconnected and interdependent (31). Under traditional conditions, spatial and informational fragmentation has impeded effective coordination among chain actors, rendering the system acutely vulnerable to external shocks (32). The adoption of digital technologies enables agricultural firms to extensively connect factors, processes and participants across the chain, dismantling information silos and organizational boundaries. This connectivity reshapes the chain from a loose, linear structure toward a tighter, more networked configuration, thereby reinforcing its structural stability against external disruptions. For food security, this stability is critical, as it ensures supply continuity and directly safeguards the stability dimension of food availability and access.

Second, digital technology innovation is highly replicable and, as an information good, non-rivalrous in use. Traditional production factors such as land and capital are exclusive in use, hindering real-time coordination across multiple actors or segments. In contrast, data generated through digital technology can be replicated and disseminated at a marginal cost approaching zero (33). This attribute means that once agricultural firms have integrated digital technology into their operations, the resulting data can be transformed into actionable information and decision-support capabilities that are simultaneously accessible to all nodes along the chain. This substantially enhances the efficiency of resource allocation for risk response and function maintenance, thereby strengthening the operational resilience of the chain under resource constraints. Such resilience is of direct relevance to food security, as it helps sustain the flow of food products and stabilize supply when resources are scarce.

Third, digital technology exhibits the ability to iterate continuously and adapt dynamically. Unlike conventional fixed equipment, digital systems are self-improving through data accumulation and algorithmic refinement (34). This self-reinforcing evolutionary dynamic shifts the industrial chain from a static technological foundation toward a more fluid and responsive configuration (35). When external conditions shift or novel risks emerge, agricultural firms can respond through software updates, parameter adjustments or platform upgrades, without requiring large-scale physical retrofitting. Such adaptability enables the chain to absorb persistent uncertainties over time, thereby bolstering long-term resilience. This resilience, in turn, underpins food security by enabling supply chains to maintain operational continuity and respond nimbly to evolving external circumstances. Therefore, this paper proposes Hypothesis 1:

*H1*: Digital technology innovation enhances agricultural chain resilience and contributes to food security.

### 2.2 Mechanisms linking digital technology innovation to agricultural industry chain resilience

Operational efficiency captures the technological dividend achieved through process optimization, whereas market expectations represent the informational dividend derived from improved foresight. Relative to a single-mediator approach, this dual-channel framework provides a more complete understanding of the resilience-building process, as it simultaneously accounts for the ability to manage disruptions through operational agility and the ability to maintain supply stability through expectational coordination. Consequently, the mediation analysis offers direct implications for food security along two distinct dimensions: short-term operational stability and long-term supply predictability.

#### 2.2.1 The mediating role of operational efficiency in food security

Digital technology innovation enhances agricultural industry chain resilience by improving two distinct dimensions of firm operational efficiency. These dimensions are internal capital efficiency and external capital efficiency.

Internal capital efficiency captures the speed and effectiveness of a firm in converting current assets into sales revenue (36). Digital technology innovation, particularly IoT-enabled inventory tracking and AI-driven demand forecasting, mitigates information frictions and improves coordination across procurement, storage and sales. By accelerating internal capital circulation, these technologies reduce inventory holding periods (37). This accelerated turnover helps curb spoilage rates and enables firms to maintain adequate buffer stocks, thereby reducing their vulnerability to supply disruptions. Viewed from this perspective, internal capital efficiency constitutes a positive mediating mechanism by which digital technology innovation strengthens agricultural chain resilience, with notable implications for food security.

External capital efficiency pertains to the management of downstream credit relationships, as reflected in the speed of customer payment collection (38). These technologies, particularly blockchain-based smart contracts and automated credit scoring, ease information asymmetry and diminish the enforcement costs inherent in credit transactions. These improvements allow agricultural firms to extend strategic credit to downstream partners. Such implicit working capital transfers stabilize the cash flow of downstream entities, thereby securing continuous food procurement and reinforcing the resilience of downstream operations. In addition, accounts receivable serve as a risk-sharing mechanism between core firms and their downstream partners (39). By adjusting payment terms in response to shocks, core firms with sufficient financial slack can absorb liquidity disruptions and prevent localized defaults from escalating into chain-wide failures. Thus, external capital efficiency constitutes a positive mediating channel through which digital technology innovation reinforces agricultural industry chain resilience, with demonstrable implications for food security. Therefore, this paper the following Hypothesis:

*H2a*: Digital technology innovation strengthens agricultural chain resilience through improved internal capital efficiency.

*H2b*: Digital technology innovation strengthens agricultural chain resilience through improved external capital efficiency.

#### 2.2.2 The mediating role of market expectation in food security

Digital technology innovation shapes capital market expectations toward agricultural firms. By sharpening demand forecasts, enabling real-time traceability, and signaling commitment to sustainable operations, it reduces the information gap between management and outside investors. Investors respond by marking up future earnings prospects and marking down the perceived risk of agricultural exposures, reassessing firm quality upward in light of these digital capabilities. These expectation shifts feed directly into higher market capitalization.

As a first-order consequence, a larger market capitalization lowers the cost of equity and widens access to debt finance through improved credit ratings (40). The resulting financial headroom allows firms to invest in resilience-enhancing assets that would otherwise be out of reach, with priority given to cold storage, real-time monitoring systems, and diversified sourcing networks. These assets cut post-harvest losses, extend shelf life, and create shock absorbers. Consequently, better-capitalized firms are in a stronger position to preserve food supply continuity when disruptions hit.

Beyond these financial channels, a high market capitalization also strengthens relational coordination across the chain. Downstream distributors and retailers read high valuation as a signal of financial soundness and long-term staying power (41), given that any failure to honor commitments would impose substantial reputational and market penalties on a highly visible firm. This makes them more willing to sign long-term procurement agreements. Upstream farmers and cooperatives, typically constrained by tight liquidity, become more willing to align their production plans with a highly valued buyer, trusting that payment and off-take promises will be kept. In turn, this trust-based coordination trims transaction costs and narrows supply uncertainty, thereby protecting the steady movement of food from farm to fork.

Furthermore, beyond improving access to capital and strengthening relational coordination, a high market capitalization affords strategic room for maneuver during systemic shocks (42). Specifically, when extreme weather, trade policy shifts, or pandemic-induced logistics failures hit, a highly valued agricultural firm can raise emergency funding on favorable terms through seasoned equity offerings or convertible debt. The swiftly raised funds can be channeled into alternative sourcing, subsidizing inventory holding for downstream partners, or absorbing price spikes that would otherwise fracture the chain. Thus, market capitalization converts collective market sentiment into a deployable financial cushion, insulating the agricultural chain from cascading breakdowns.

Based on the above reasoning, we propose the following hypothesis.

*H3*: Digital technology innovation in agricultural firms enhances agricultural industry chain resilience through the mediating role of market capitalization.

### 2.3 Heterogeneous effects across regions and firm types

The enabling effect of digital technology innovation in agricultural firms on agricultural industrial chain resilience is not a homogeneous or universal process; rather, it is structurally moderated by regional institutional environments, industrial ecological conditions, and firms’ own endowments (43, 44).

From a regional perspective, drawing on technology diffusion theory and regional innovation system theory, the realization of digital technology’s value is highly contingent on the absorptive capacity and transformation conditions of the socio-economic system in which it is embedded (45). Different regions exhibit pronounced gradient disparities in digital infrastructure levels, institutional environment quality, industrial ecosystem maturity, and financial resource accessibility. These structural factors determine whether firm-level digital technology innovation can translate into systemic agricultural industrial chain resilience, a capability that is essential for maintaining stable food supply, ensuring continuous food availability, and withstanding disruptions that directly threaten food security.

From a firm-level perspective, drawing on the resource-based view and organizational learning theory, the effectiveness of digital technology innovation is likewise shaped by firms’ own resource endowments, governance structures, organizational age, and credit accumulation (46). These firm-specific characteristics influence whether digital innovation translates into operational efficiency and financial stability, which in turn shapes the firm’s capacity to secure reliable procurement, storage, and distribution of agricultural products along the chain. Therefore, this paper proposes Hypothesis 4:

*H4*: The effect of digital technology innovation on agricultural chain resilience is heterogeneous across regions and firm types.

**Figure 2:**
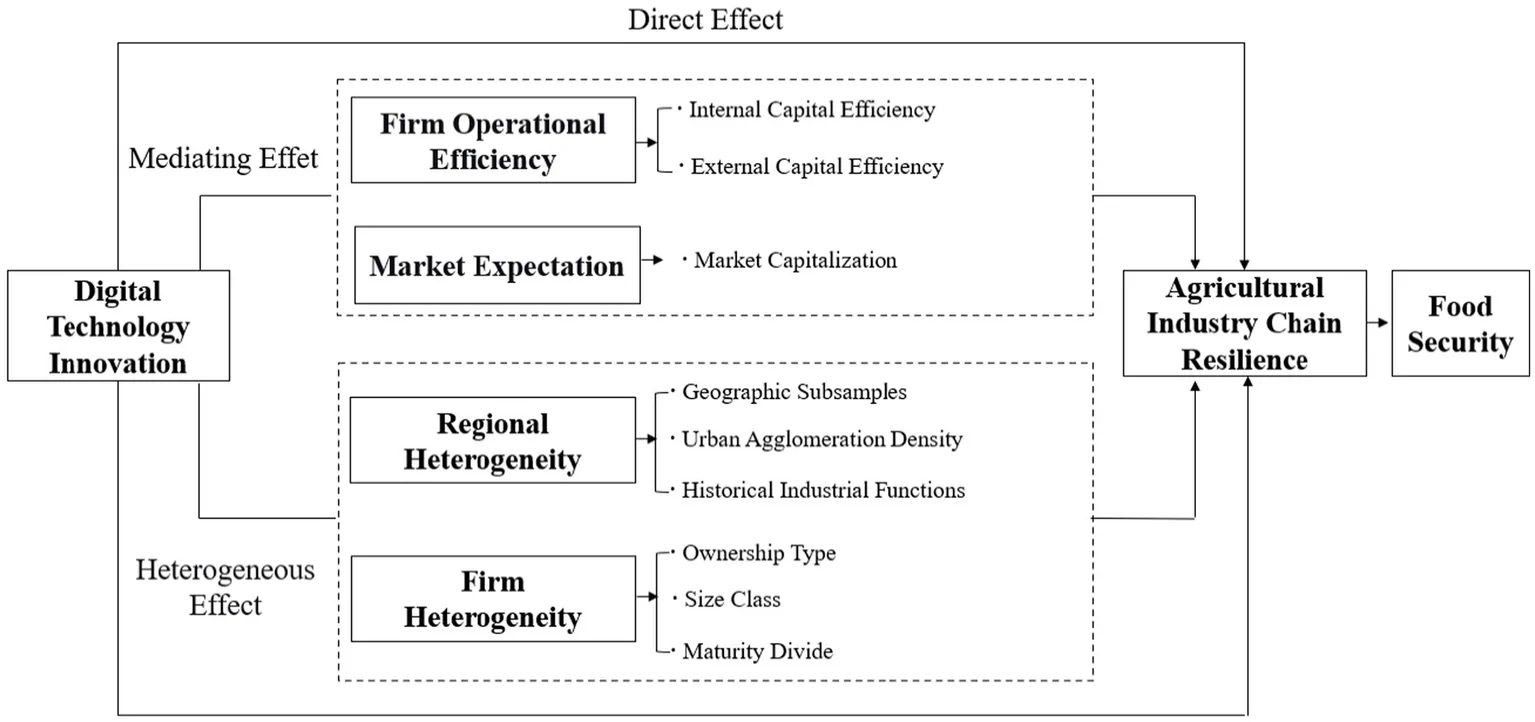
Theoretical model diagram.

The theoretical model diagram of the above research hypotheses is shown in Figure 2.

## 3 Research design

### 3.1 Empirical model

To empirically examine the impact of digital technology innovation in agricultural firms on agricultural industrial chain resilience, this paper employs a two-way fixed effects model, specified in Equation 1 below:


$${\text{Aicr}}_{\mathrm{it}}=\alpha_{0}+\alpha_{1}\mathrm{Dtf}\_{\text{Agri}}_{\mathrm{it}}+\varphi {\text{Controls}}_{\mathrm{it}}+{\text{Firm}}_{\mathrm{it}}+{\text{Year}}_{\mathrm{it}}+\varepsilon_{\mathrm{it}}$$ (1)

where i and t index firms and years, respectively; $\mathrm{Aic}r_{\mathrm{it}}$ denotes the industrial chain resilience of firm i in year t; $\mathrm{Dtf}\_\mathrm{Agr}i_{\mathrm{it}}$ is the core explanatory variable, measured by the frequency of digital-technology-related terms; $\text{Control}s_{\mathrm{it}}$ represents firm-level control variables; $\mathrm{Fir}m_{\mathrm{it}}$ captures firm fixed effects; $\mathrm{Yea}r_{\mathrm{it}}$ *c*aptures year fixed effects; and $\varepsilon_{\mathrm{it}}$ is the random error term.

To test the transmission mechanisms, this paper employs the stepwise mediation analysis and constructs a recursive model, as specified in Equation 2.


$$M_{\mathrm{it}}=\beta_{0}+\beta_{1}\mathrm{Dtf}\_{\text{Agri}}_{\mathrm{it}}+\varphi {\text{Controls}}_{\mathrm{it}}+{\text{Firm}}_{\mathrm{it}}+{\text{Year}}_{t}+\varepsilon_{\mathrm{it}}$$ (2)

where $M_{\mathrm{it}}$ denotes the mediating variables.

### 3.2 Variables selection

#### 3.2.1 Explained variable

Agricultural industry chain resilience (Aicr): this composite indicator captures customer structure dispersion and supply chain concentration risk, characterizing the agricultural chain’s stability and recovery capacity under external shocks (47). Greater customer dispersion weakens shock transmission from any single customer, implying stronger resilience. The construction of Aicr relies on two negative indicators, namely customer dependence, defined as the share of sales to the top five customers, and the Herfindahl–Hirschman Index of customer concentration. Each is min-max normalized, and the arithmetic mean of the normalized values is then reverse-transformed (1-mean) to obtain Aicr. A larger Aicr signifies a more dispersed customer base, lower concentration risk, and greater chain resilience. By diversifying the customer portfolio, a higher Aicr stabilizes distribution networks and prevents localized demand shocks from cascading into systemic supply failures, thereby reinforcing food availability and overall food security.

#### 3.2.2 Core explanatory variable

Digital technology innovation in agricultural firms (Dtf_Agri): digital technology innovation in agricultural firms is the core driver of the digital transformation of agricultural industrial chains. By embedding digital technologies—such as big data, artificial intelligence, and the Internet of Things—into agricultural production, processing, distribution, and marketing, digital technology innovation can significantly enhance the resource allocation efficiency of agricultural firms, reduce information asymmetry between upstream and downstream segments along the chain, and foster new business forms and models. This paper employs the technological innovation word frequency indicator from the CSMAR database as a proxy for firms’ digital technology innovation (48). Constructed from the annual reports of listed companies, this indicator counts the frequency of keywords associated with digital technology and directly captures the intensity of innovation input and the level of digital technology application. A larger value of this indicator signifies a higher level of digital technology innovation in firms. This word-frequency indicator is preferred over patent-based measures chiefly because it better captures firms’ overall digital technology deployment from annual report disclosures, whereas patents reflect only inventive outputs and largely overlook technology adoption and application.

#### 3.2.3 Mediator variables

Internal capital efficiency (Catr): this variable is measured by the current asset turnover ratio, calculated as operating revenue divided by average total current assets. A higher value reflects stronger revenue generation with limited current assets, indicating proficiency in capital coordination across internal fund allocation and operational circulation, including cash utilization, inventory turnover, accounts receivable collection, and short-term investment liquidity. The current asset turnover ratio thus serves as both a core indicator of asset efficiency and a micro-level operational basis for agricultural industrial chain resilience. By shortening inventory holding periods and accelerating capital recirculation, improved internal capital efficiency reduces perishable losses and facilitates timely market release of stored outputs, containing waste at the source and reinforcing food supply continuity.

External capital efficiency (Artr): we proxy for this variable using the accounts receivable turnover ratio, calculated as operating revenue divided by average accounts receivable (49). The ratio reflects the speed of credit collection from downstream customers, with faster collection indicating less capital tied up downstream. This speed indirectly captures a firm’s credit management capability, bargaining power, credit policy rigor, payment enforcement efficiency, and bad debt prevention. A higher turnover ratio accelerates cash recovery and frees working capital for ongoing procurement and distribution. This strengthened financial footing reduces the risk of payment-driven disruptions downstream and helps maintain stable food flows across the chain.

Market expectation (lnMc): the natural logarithm of market capitalization proxies for this variable. A higher value signals stronger market confidence in the firm’s future performance, reflecting expectations of efficiency gains, strategic adaptability, and sustainable growth enabled by digital innovation. Enhanced market valuation improves external financing access by lowering the cost of capital and strengthening credit standing. This financial headroom buffers against liquidity shocks, allowing firms to preserve procurement volumes and distribution schedules during crises, thereby protecting food availability for end consumers.

#### 3.2.4 Control variables

The control variables are as follows:

Leverage ratio (Lev): it is specifically measured as the ratio of total liabilities to total assets (50). A higher share of liabilities subjects the firm to greater debt pressure and operational uncertainty, which can constrain its ability to maintain stable procurement and distribution activities when external shocks occur, thereby weakening supply chain continuity and undermining food security.

Firm size (lnSize): firm size captures a firm’s resource endowments and risk-bearing capacity and is measured as the natural logarithm of total assets (51). Larger firms typically possess greater capital reserves, more developed organizational structures, and stronger market bargaining power, enabling them to absorb the fixed costs of digital transformation and withstand technological uncertainties. Consequently, greater firm size provides a stable operational base that buffers the industrial chain against demand and supply fluctuations, contributing to the reliability of food supply.

Ownership concentration (Oc): this indicator reflects the degree of concentration in a firm’s equity structure and its associated governance characteristics (52). A higher value enables faster decision-making, facilitating rapid supply chain adjustments under external shocks. However, excessive concentration may also induce tunneling behaviors, such as related-party transactions or capital occupation, that deplete corporate resources. Given this duality, the effect of ownership concentration on chain resilience is non-linear, with moderate concentration supporting coordinated crisis response and excessive control eroding the financial buffer that underpins sustained food distribution during disruptions.

Fixed asset ratio (Far): measured as fixed assets divided by total assets, this variable captures the physicality of a firm’s asset structure and its resource allocation pattern (53). A higher ratio generally indicates greater investment in plants, equipment, warehousing, and logistics infrastructure, which enhances the physical reliability of storage and transportation networks. In turn, this asset-backed stability ensures that food can be preserved and moved along the chain even under stress, directly reinforcing the system’s capacity to safeguard food security.

### 3.3 Data description and descriptive statistics

Given the availability and completeness of financial data for listed agricultural firms, this study adopts 2010–2025 as the sample period. The original data are drawn from the CSMAR database and the annual reports of listed companies. To mitigate the potential influence of anomalous observations on the estimation results, we exclude firm-year observations of companies designated as ST (special treatment) or *ST (delisting risk warning) and those with abnormal financial data, yielding a final sample of 714 firm-year unbalanced panel observations from 69 listed agricultural firms. Given that the sample consists exclusively of publicly listed firms, interpretative caution is warranted when generalizing the findings to the broader population of agricultural firms in China. Table 1 reports the descriptive statistics of the main variables.

**Table 1:** Description of variables.

Variable type	Variable name	Symbol	Obs	Mean	SD	Min	Max
Explained variable	Agricultural industry chain resilience	Aicr	714	0.8790	0.1383	0.2868	0.9958
Core explanatory variable	Digital technology innovation in agricultural firms	Dtf_Agri	714	0.8246	0.9991	0.0000	4.1271
Mediator variables	Internal capital efficiency	Catr	433	1.2986	1.0419	0.0978	5.7122
	External capital efficiency	Artr	714	0.1407	0.1413	0.0000	0.5452
	Market expectation	lnMc	517	22.6599	1.0207	20.9503	26.3253
Control variables	Leverage ratio	Lev	714	0.4904	0.2215	0.0577	1.0848
	Firm size	lnSize	714	21.7737	1.1289	19.4321	25.2780
	Ownership concentration	Oc	714	30.9285	14.1003	3.9664	70.3173
	Fixed asset ratio	Far	714	0.2761	0.1674	0.0072	0.6757

## 4 Empirical analysis

### 4.1 Benchmark regression analysis

The analysis focuses on column (5) of Table 2, which includes the full set of control variables and fixed effects. The results indicate that the coefficient of digital technology innovation is significantly positive, thereby supporting Hypothesis 1. This conclusion is consistent with the findings of Dang et al. (54) and Bekele et al. (55), who, respectively, demonstrate that larger firms possess greater resource accessibility and that firm size distribution facilitates digital lending, both supporting the resilience-enhancing role of digital technology innovation. This effect is theoretically consistent with endogenous growth theory, wherein digital technologies mitigate information frictions and coordination costs, thereby strengthening the system’s adaptive capacity. From a food security standpoint, the underlying logic lies in the fact that, by being embedded into the full chain of agricultural production, processing, distribution, and sales, digital technology significantly enhances information transmission efficiency and resource allocation capacity, thereby strengthening the chain’s buffer capacity and recovery elasticity in the face of external shocks that could otherwise disrupt food supply and compromise food availability.

**Table 2:** Benchmark regression results.

Variable	(1)	(2)	(3)	(4)	(5)
Dtf_Agri	0.0161** (0.0078)	0.0166** (0.0078)	0.0147* (0.0080)	0.0148* (0.0077)	0.0147* (0.0078)
Lev		0.1104** (0.0454)	0.0976** (0.0440)	0.1020** (0.0427)	0.1028** (0.0423)
lnSize			0.0113 (0.0103)	0.0104 (0.0102)	0.0107 (0.0101)
Oc				0.0012 (0.0011)	0.0012 (0.0011)
Far					−0.0116 (0.0697)
Cons	0.7952*** (0.0279)	0.7478*** (0.0327)	0.5180** (0.2121)	0.4897** (0.2186)	0.4879** (0.2195)
Firm fixed	Yes	Yes	Yes	Yes	Yes
Year fixed	Yes	Yes	Yes	Yes	Yes
Observations	714	714	714	714	714
R^2^	0.2387	0.2611	0.2641	0.2693	0.2694

*, ** and ***, respectively, indicate significance at the 10, 5 and 1% levels respectively, with standard errors of the estimated coefficients in parentheses.

Specifically, on the one hand, digital technology innovation reduces the degree of information asymmetry among upstream and downstream entities along the chain and optimizes supply demand matching and inventory management, which helps prevent food shortages and reduces postharvest losses. On the other hand, it raises the level of standardization and traceability in production processes, reinforcing the supply chain’s ability to withstand disruptions and ensuring that safe and stable food supplies reach consumers even during crises. Therefore, digital technology innovation in agricultural firms constitutes not only a vital pathway for enhancing their own competitiveness but also a key driving force for consolidating the foundations of agricultural industrial chain resilience from the micro level, thereby supporting the conditions necessary for long term food security.

### 4.2 Robustness checks

#### 4.2.1 Variable substitution

The level of digital technology application in agricultural firms is adopted as a proxy for their digital technology innovation. This indicator directly captures the depth of adoption and intensity of use of digital infrastructure, information systems, and smart terminals, consistent with the fundamental logic in the economics of technological innovation that technology adoption constitutes the realization of innovation (56). As shown in column (1) of Table 3, after replacing the core explanatory variable, the coefficient of digital technology innovation in agricultural firms remains positive and statistically significant, with both its direction and significance consistent with the baseline model. These results indicate that the research findings are robust.

**Table 3:** Robustness checks.

Variable	(1) Variable substitution	(2) Omitting top-tier firms	(3) Omitting municipalities	(4) Alternative estimator	(5) Lagged digital innovation
Dtf_Agri	0.0020** (0.0010)	0.0147** (0.0074)	0.0271*** (0.0098)	0.0145^**^ (0.0071)	
L. Dtf_Agri					0.0111* (0.0059)
Lev	0.0927^**^ (0.0379)	0.0472 (0.0406)	0.1085** (0.0448)	0.0552 (0.0392)	0.0903* (0.0468)
lnSize	0.0157^*^ (0.0079)	0.0162* (0.0095)	0.0259** (0.0115)	0.0154^*^ (0.0083)	0.0175* (0.0096)
Oc	−0.0011 (0.0012)	0.0004 (0.0009)	0.0003 (0.0011)	0.0006 (0.0009)	0.0010 (0.0011)
Far	−0.0285 (0.0871)	0.0309 (0.0630)	−0.0504 (0.0836)	0.0201 (0.0605)	−0.0295 (0.0796)
Cons	0.5230^***^ (0.1842)	0.3928* (0.2077)	0.2440 (0.2495)	0.4123^**^ (0.1836)	0.5180** (0.2121)
Firm fixed	Yes	Yes	Yes	Yes	Yes
Year fixed	Yes	Yes	Yes	Yes	Yes
Observations	577	657	640	714	620
R^2^	0.1464	0.2685	0.1401	0.2641	0.2649

*, ** and ***, respectively, indicate significance at the 10, 5 and 1% levels respectively, with standard errors of the estimated coefficients in parentheses.

#### 4.2.2 Omitting top-tier firms

To ensure that the research findings are not driven by idiosyncratic factors specific to the agricultural sector, a robustness check is conducted by removing representative firms that are large in scale and highly concentrated in their business operations along the agricultural industrial chain. Specifically, the excluded sample comprises COFCO Sugar (600737), Beidahuang (600598), New Hope (000876), Muyuan Foods (002714), Wens Foodstuff (300498), Zhengbang Technology (002157), Dabeinong (002385), and Longping High-Tech (002998). As confirmed by the regression results in column (2) of Table 3, after re-estimation, the sign and statistical significance of the coefficient on digital technology innovation remain consistent with those of the baseline regression, providing further evidence that the main findings are robust.

#### 4.2.3 Omitting municipalities

To rule out the potential interference of the endowment advantages enjoyed by directly administered municipalities—in terms of policy support, financing access, and management efficiency—due to their higher administrative rank, a robustness check is performed by excluding the samples of Beijing, Shanghai, Tianjin, and Chongqing. As revealed in column (3) of Table 3, after excluding these municipalities, the estimates of the core explanatory variable exhibit no substantive change, from which the robustness of the research findings is obtained.

#### 4.2.4 Alternative estimator

Replacing the econometric estimation method constitutes a standard approach in robustness checks. A random effects model is employed to re-estimate the regression. As demonstrated by the results in column (4) of Table 3, the estimated effect of digital technology innovation in agricultural firms on agricultural industrial chain resilience remains positive. This finding proves that the choice of estimation method does not alter the sign or statistical significance of the coefficient, and that the research findings are highly robust.

#### 4.2.5 Lagged digital innovation

To mitigate potential endogeneity arising from reverse causality, the core explanatory variable is lagged by one period. As indicated by the regression results in column (5) of Table 3, the sign and statistical significance of the coefficient on the one-period-lagged digital technology innovation in agricultural firms remain consistent with those of the baseline regression. This finding suggests that the core conclusions do not hinge on the contemporaneous effect of the variable, thereby further reinforcing the robustness of the research findings.

### 4.3 Mediation analysis

#### 4.3.1 Internal capital efficiency as mediator

As shown in column (1) of Table 4, digital technology innovation in agricultural firms has a significant positive effect on the current asset turnover ratio (Catr), a proxy for internal capital efficiency. To further explore the underlying mediation mechanism, we conducted a bootstrap analysis with 5,000 replications. The indirect effect of digital technology innovation on firm performance through Catr yielded a bootstrap 95% confidence interval of [0.0111, 0.1556]. Notably, this interval does not include zero, confirming that the mediation effect is statistically significant. This result echoes da Silveira et al. (57), who associate precision agriculture adoption with farm size and financial resources, and reinforces Han et al. (58), whose Chinese evidence shows that farm scale and digital capital promote both technology adoption and asset turnover efficiency. Hypothesis 2a is thus supported. Regarding corporate financial and operational optimization, once digital technology is deeply embedded in the agricultural industrial chain, firms can achieve information transparency and process automation across the entire procurement–production–sales continuum through means such as IoT monitoring, blockchain traceability, and smart contracts. Specifically, firms are able to accurately forecast market demand, optimize inventory levels, and accelerate sales settlement and fund recovery, thereby significantly improving the turnover speed and utilization efficiency of current assets while maintaining reasonable liquidity. In terms of corporate resource integration, digital technology innovation endows firms with stronger collaborative management capabilities, enabling them to reduce the dwell time of capital across different segments of the chain and reallocate the freed-up liquidity to high-return productive activities, thus creating a virtuous cycle of efficiency gains, capital release, and reinvestment. It follows that the marked improvement in current asset turnover efficiency represents a positive outcome of the intelligent transformation of firms’ financial behavior driven by digital technology innovation.

**Table 4:** Testing of mediation effects.

Variable	(1) Catr	(2) Artr	(3) lnMc
Dtf_Agri	0.0833* (0.0421)	0.0188* (0.0094)	0.0493** (0.0243)
Cons	6.2740*** (2.1610)	0.8267*** (0.2581)	7.2354*** (0.6494)
Controls	Yes	Yes	Yes
Firm fixed	Yes	Yes	Yes
Year fixed	Yes	Yes	Yes
Observations	433	714	516
R^2^	0.1340	0.0797	0.5760

*, ** and ***, respectively, indicate significance at the 10, 5 and 1% levels respectively, with standard errors of the estimated coefficients in parentheses.

From a food security perspective, the improvement in current asset turnover efficiency strengthens industrial chain resilience (59). From an operational standpoint, a faster rate of capital recovery provides core firms with greater financial buffer room when facing sudden shocks, effectively guarding against liquidity crises triggered by abrupt changes in the external environment. More importantly, faster turnover of current assets directly reduces the time that perishable agricultural products remain in storage, thereby minimizing spoilage and postharvest losses. This acceleration ensures that food moves more quickly from producers to consumers, reducing the risk of supply interruptions and stabilizing food availability during seasonal or climate induced shocks. At the same time, a highly efficient turnover model helps firms reduce inefficient capital lock-up across production and operational stages and, by leveraging flexible collection mechanisms, enhances their capacity to extend credit support to upstream and downstream partners, thereby ensuring the continuous functioning of the transaction chain amid fluctuations.

In sum, the Catr serves as a positive mediator between digital technology innovation and agricultural industrial chain resilience. Through enhancing capital operation efficiency, digital technology strengthens the intrinsic stability of the industrial chain in terms of resource allocation and risk response, which directly contributes to safeguarding food security by maintaining continuous food supply and reducing postharvest losses. Hypothesis 2 is thus supported.

#### 4.3.2 External capital efficiency as mediator

As shown in column (2) of Table 4, digital technology innovation in agricultural firms significantly enhances the accounts receivable turnover ratio (Artr), which serves as a measure of external capital efficiency. This finding is corroborated by Qiao et al. (60) and Yu et al. (61), who, respectively, document that digital economy development improves factor allocation efficiency, and show that supply chain digitalization mitigates investment mismatch and accelerates receivable turnover. Collectively, these results lend strong support to Hypothesis 2b. Drawing on information asymmetry theory and the resource-based view, digital technology reduces the costs firms incur in identifying the contract performance capacity of downstream customers and in monitoring credit transactions. The alleviation of information constraints enables firms to redefine accounts receivable from a passive risk exposure to a strategic credit resource that can be actively managed, thereby selectively extending payment terms or raising credit lines for high quality downstream entities to lock in cooperative relationships and build exclusive channel networks. Financially, this behavior manifests as a compliant increase in net accounts receivable, driven not by deteriorating customer payment capacity but by proactive credit expansion powered by digital risk control capabilities.

In the context of food security, a moderate increase in the Artr positively influences agricultural industrial chain resilience. Specifically, the accounts receivable accumulated by core firms constitute an implicit liquidity injection into downstream partners, easing their financing constraints, reducing the vulnerability of individual operations, and preventing chain wide ruptures. This enhanced cash flow stability is critical for food security, as it enables downstream distributors and retailers to maintain continuous food procurement and hold adequate inventory even during credit squeezes or liquidity shortages. By preventing disruptions in inventory replenishment, the Artr mechanism ensures that food products remain available along the supply chain. At the same time, the incremental accounts receivable embed informal risk sharing contracts. When negative shocks strike, core firms can use the stock of accounts receivable as a buffer, flexibly adjust payment terms, and proactively absorb short term losses, thereby shifting the industrial chain from a loosely coupled configuration based on cash on delivery toward a tightly symbiotic structure characterized by credit interlocking and risk sharing. This transformation directly safeguards food security by maintaining the flow of food from producers to consumers without interruption, even in the face of external shocks such as trade frictions, extreme weather events, or public health crises.

#### 4.3.3 Market expectation as mediator

Digital technology innovation in agricultural firms is found to significantly and positively influence market expectation, which is proxied by the natural logarithm of market capitalization (lnMc) and serves as the mediating variable linking digital innovation to the resilience of the agricultural industry chain, as presented in column (3) of Table 4. As shown in column (3) of Table 4, digital technology innovation in agricultural firms significantly enhances the accounts receivable turnover ratio (Artr), which serves as a measure of external capital efficiency. This finding aligns with the conclusions of Bharadwaj et al. (62) and Froot et al. (63). Accordingly, Hypothesis 3 receives strong empirical support. Drawing on signaling theory and adaptive market hypothesis, digital technology reduces the costs firms incur in collecting, processing, and disseminating market intelligence. The alleviation of information asymmetry enables agricultural enterprises to form more accurate and forward-looking market expectations, thereby shifting from reactive production planning to proactive capacity allocation based on anticipated demand signals. Economically, this behavior manifests as a measurable increase in market expectation indices, driven not by speculative sentiment but by data-enhanced predictive capabilities.

In food security terms, rising market expectations contribute meaningfully to strengthening the resilience of the agricultural industry chain. Specifically, improved market expectations lower uncertainty across the chain, prompting upstream producers to stabilize planting decisions and downstream distributors to uphold optimal inventory levels. This expectation-driven coordination mitigates the bullwhip effect and prevents the simultaneous crises of overproduction and shortage. By aligning supply with anticipated demand, the market expectation mechanism ensures a steady flow of food products along the supply chain. Moreover, elevated market expectations embed implicit coordination protocols among chain participants. In the event of external shocks such as extreme weather or trade restrictions, convergent expectations allow for rapid and synchronized adjustments in production, storage, and distribution, obviating costly renegotiation or panic stocking. This reconfigures the agricultural industry chain from a fragile and reactive structure into an expectation-coordinated, resilient framework that sustains food availability under disruption. Hence, through the mediating channel of market expectations, digital technological innovation directly reinforces food security by safeguarding both the integrity and continuity of food supply during regular operations and disruptive episodes alike.

## 5 Heterogeneity analysis

### 5.1 Regional heterogeneity

To examine the regional heterogeneity in the impact of digital technology on agricultural industrial chain resilience, this paper conducts tests along three dimensions: macro-regional patterns, urban agglomeration density, and historical industrial functions. Specifically, in accordance with the regional classification scheme of *the National Bureau of Statistics*, the provincial-level administrative units nationwide are divided into eastern, central, and western regions based on geographic location and economic development patterns. Similarly, based on the 19 state-level urban agglomerations delineated in *the National Plan for New-Type Urbanization (2014–2020)*, cities are distinguished by whether they are covered by any designated urban agglomeration plan. Likewise, drawing on the list published in *the National Plan for the Adjustment and Transformation of Old Industrial Bases (2013–2022)*, a list-based identification approach is adopted, whereby cities included in the plan are classified as old industrial base cities and the remainder as non-old industrial base cities.

#### 5.1.1 Geographic subsamples

The results reported in columns (1) to (3) of Table 5 show that the positive effect of digital technology innovation in agricultural firms on industrial chain resilience is concentrated and statistically significant only in the eastern region, while it is not significant in the central and western regions. Drawing on technology diffusion theory and regional innovation system theory, this regional heterogeneity arises from the structural disparities among the three regions in terms of digital infrastructure, institutional environment, industrial ecology, and financial resources. The eastern region, by virtue of its advanced digital infrastructure, well developed supply chain coordination systems, vibrant financial markets, and first trial policy advantages, has formed a complete closed loop ecosystem that translates firm level technological innovation into systemic resilience (64). From a food security perspective, this resilience advantage in the eastern region contributes to more stable food supply chains and a greater capacity to maintain food availability during external shocks.

**Table 5:** Regression results of regional heterogeneity analysis.

Variable	(1) Eastern region	(2) Central region	(3) Western region	(4) Urban agglomeration	(5) Non-urban agglomeration area	(6) Non-old industrial base	(7) Old industrial base
Dtf_Agri	0.0186* (0.0101)	0.0107 (0.0065)	0.0094 (0.0127)	0.0169* (0.0092)	0.0035 (0.0117)	0.0147* (0.0081)	−0.0055 (0.0053)
Cons	0.3764 (0.2533)	0.6004** (0.2371)	−0.0301 (0.6459)	0.4253 (0.2806)	1.1828* (0.5519)	0.5267** (0.2371)	0.8703** (0.2350)
Controls	Yes	Yes	Yes	Yes	Yes	Yes	Yes
Firm fixed	Yes	Yes	Yes	Yes	Yes	Yes	Yes
Year fixed	Yes	Yes	Yes	Yes	Yes	Yes	Yes
Observations	397	160	157	575	139	670	44
R^2^	0.3314	0.3962	0.2999	0.2863	0.2459	0.2648	0.9203

*, ** and ***, respectively, indicate significance at the 10, 5 and 1% levels respectively, with standard errors of the estimated coefficients in parentheses.

In contrast, the central region is constrained by uneven digital infrastructure coverage, a relatively low degree of marketization of data factors, and insufficient technology intermediary capacity, which limits the transmission efficiency of technological outcomes. The western region, due to geographic dispersion, the small scale of business entities, high costs of digital application, and pronounced financial exclusion, sees digital technology application largely confined to localized optimization within production processes, making it difficult to reach the systemic restructuring of capital flows, credit flows, and information flows along the industrial chain. Consequently, the central and western regions remain vulnerable links in the food system, where limited digital empowerment heightens the risk of food supply disruptions.

#### 5.1.2 Urban agglomeration density

The results reported in columns (4) and (5) of Table 5 show that the positive enabling effect of digital technology innovation in agricultural firms on industrial chain resilience is concentrated in the urban agglomeration sample, while it is not significant in non-urban agglomeration areas. This can be attributed to the fact that within urban agglomerations, the high-density agglomeration of firms, well developed logistics and information infrastructure, and close industrial linkages enable digital technology innovation to form a highly efficient transformation pathway from individual firm level actions to systemic industrial chain resilience through rapid credit circulation, risk sharing, and coordinated response mechanisms (65). This transformation pathway in urban agglomerations therefore enhances food supply chain stability and supports the maintenance of food availability during systemic shocks.

In contrast, non-urban agglomeration areas tend to be characterized by dispersed business entities, limited access to public services, a shortage of financial and intermediary services, and relatively small market scale. Under these conditions, the application of digital technology often remains confined to efficiency improvements within isolated segments, falling short of triggering a systemic reconfiguration of upstream and downstream capital flows, credit flows, and information flows. As a result, food supply chains in these regions are less likely to benefit from resilience gains, leaving them more exposed to disruptions when shocks occur.

#### 5.1.3 Historical industrial functions

The results reported in columns (6) and (7) of Table 5 show that the positive enabling effect of digital technology innovation in agricultural firms on industrial chain resilience is statistically significant in the non-old industrial base sample, while it is not significant in the old industrial base sample. Drawing on path dependence theory and the regional lock-in effect framework, this heterogeneity can be attributed to the systematic differences between old and non-old industrial bases in terms of industrial structural rigidity, institutional inertia, innovation ecosystem vitality, and the degree of financial marketization. Non-old industrial bases are predominantly driven by emerging industries, feature diverse market entities, and exhibit a high penetration rate of digital technology. Their flexible industrial organization and open credit environment enable digital technology innovation to efficiently drive supply chain finance, collaborative governance, and risk-sharing mechanisms, thereby forming a smooth transmission pathway from firm-level technology application to the enhancement of industrial chain resilience. In terms of food security, this resilience advantage in non-old industrial bases translates into more stable food supply chains, reduced risks of disruption, and greater capacity to maintain food availability during external shocks.

Meanwhile, old industrial bases have long been dominated by heavy and chemical industries, with a relatively high share of the state-owned economy, substantial accumulated historical sunk costs, and lagging digital infrastructure upgrading. These regions also suffer from inadequate digital foundations, and the capital formation mechanisms in agriculture-related sectors have yet to adapt fully to the needs of emerging business forms. As a consequence, these areas are less likely to achieve significant resilience gains, leaving their food supply chains more vulnerable to disruptions and rendering them persistent weak links in the national food security system.

### 5.2 Firm heterogeneity

To examine the heterogeneous effects of ownership structure, firm size, and life cycle stage on the relevant economic behaviors, this paper conducts subsample tests along three dimensions. Starting with exogenous ownership attributes, firms are classified as state owned enterprises (SOEs) or non-state-owned enterprises (non-SOEs) based on the nature of the ultimate controller. Moving to firm size, the sample median of total assets serves as the partition threshold, whereby firms above the median are defined as large scale enterprises and those at or below the median as small scale enterprises. Turning to endogenous temporal dynamics, a 20-year threshold since establishment is applied, with firms aged less than 20 years defined as growth stage enterprises and those aged 20 years or more as mature enterprises.

#### 5.2.1 Ownership type

The results reported in columns (1) and (2) of Table 6 show that digital technology innovation significantly enhances industrial chain resilience among non-SOEs, but not among SOEs. This ownership-based heterogeneity sheds light on the institutional conditions under which digital innovation may or may not strengthen agricultural supply chains against exogenous disruptions, a capacity central to food security.

**Table 6:** Regression results of firm heterogeneity analysis.

Variable	(1) SOEs	(2) Non-SOEs	(3) Large-scale	(4) Small-scale	(5) Growth-stage	(6) Mature
Dtf_Agri	0.0075 (0.0124)	0.0221*** (0.0078)	0.0113** (0.0056)	0.0096 (0.0123)	−0.0188 (0.0168)	0.0190** (0.0079)
Cons	0.0170 (0.5023)	0.7370*** (0.2561)	0.6890*** (0.2124)	0.2317 (0.6423)	−0.3391 (0.3685)	0.5024** (0.2474)
Controls	Yes	Yes	Yes	Yes	Yes	Yes
Firm fixed	Yes	Yes	Yes	Yes	Yes	Yes
Year fixed	Yes	Yes	Yes	Yes	Yes	Yes
Observations	251	460	357	357	51	663
R^2^	0.3831	0.2571	0.2142	0.2562	0.8030	0.2803

*, ** and ***, respectively, indicate significance at the 10, 5 and 1% levels respectively, with standard errors of the estimated coefficients in parentheses.

The divergence can be understood across three stages of translating technological inputs into resilience gains: investment motivation, implementation capacity, and resource constraints. At the motivational stage, non-SOEs, driven by profit maximization, are responsive to digital investment returns and tend to channel technologies toward resilience-enhancing uses such as real-time inventory coordination and adaptive scheduling. SOEs, by contrast, are oriented toward policy objectives including output stabilization and supply assurance, with performance evaluation not exclusively tied to economic efficiency. Their digital investment appears more compliance-driven than market-driven, which may weaken the impetus for resilience-oriented deployment and limit the operational adaptability needed during supply disruptions.

At the implementation stage, governance structures shape the speed and effectiveness of technology integration. Non-SOEs benefit from shorter decision chains and greater managerial discretion (66), enabling timely adoption of digital tools in supply chain management and risk warning systems. This agility supports rapid adjustments in procurement, storage, and distribution when shocks occur. SOEs, however, face multilayered principal-agent relationships, complex approval procedures, and risk accountability mechanisms that tend to discourage experimentation. These institutional constraints create structural rigidity, potentially delaying digital responses to emerging vulnerabilities and complicating logistics reconfiguration during emergencies.

Resource constraints add further differentiation. Non-SOEs operate under hard budget constraints that discipline investment efficiency (67), encouraging precise alignment of digital technologies with critical nodes such as demand forecasting, cold chain monitoring, and distribution routing. This amplifies the resilience gain per unit of investment. SOEs enjoy preferential access to policy-oriented credit and subsidies, softening their budget constraints. This softness reduces pressure to evaluate deployment effectiveness and may favor system acquisition over actual performance in improving supply chain continuity, so that digital investment may expand technological capacity without delivering proportionate gains in recovery capability.

#### 5.2.2 Size class

The results reported in columns (3) and (4) of Table 6 show that the positive enabling effect of digital technology innovation in agricultural firms on industrial chain resilience is statistically significant in the sample of large-scale enterprises but not significant in the sample of small-scale enterprises. This firm-size heterogeneity can be explained by three interrelated reasons.

To begin with, large-scale agricultural firms, backed by substantial financial capital, are able to bear the high fixed costs associated with deploying digital infrastructure and recruiting specialized talent. In addition, the extensive scale of their operations spanning the full chain of production, supply, and sales allows the fixed costs of digital technology to be spread thinly while marginal returns are significantly amplified (68). Furthermore, large-scale firms typically occupy core nodes in the industrial chain, commanding the convergence and distribution of credit flows, information flows, and logistics flows. As a result, they possess the systemic capacity to transform digital-technology-driven credit expansion into overall industrial chain resilience. Through this systemic capacity, large-scale enterprises reinforce food supply chain stability and help maintain food availability during market or environmental shocks.

By contrast, small-scale agricultural firms face binding constraints. Owing to limited financial resources and a thin reserve of digital talent, they often confine digital technology application to basic information recording or isolated efficiency improvements, making it difficult to establish a digital closed loop covering the entire process. Additionally, small-scale firms lack the bargaining power to extend credit or strategically empower upstream and downstream partners. Consequently, even if their own operational efficiency is improved by technology investment, they struggle to transmit such efficiency gains to the broader industrial chain system. Under these conditions, the absence of resilience gains in small-scale enterprises leaves food supply chains more susceptible to disruptions, with a higher likelihood of supply interruptions and inventory shortfalls during systemic shocks such as extreme weather events, public health emergencies, or trade frictions.

#### 5.2.3 Maturity divide

The results reported in columns (5) and (6) of Table 6 show that the positive enabling effect of digital technology innovation in agricultural firms on industrial chain resilience is statistically significant among mature enterprises but not among growth-stage enterprises. Drawing on organizational learning theory and the liability of newness framework, this heterogeneity arises from systematic differences between mature and growth-stage enterprises in their stock of credit capital, depth of relational networks, and capacity for institutionalized coordination. Mature enterprises benefit from a long-accumulated track record of contract fulfillment and reputational capital, which allows the credit expansion behaviors driven by digital technology to be effectively recognized by the market. This recognition, in turn, forges a resilience-enhancing pathway in which technological empowerment and institutional maturity work together to stabilize food supply chains and sustain food availability during disruptions.

Growth-stage enterprises face a different set of conditions. Constrained by scarce resources, a lack of credit history, and weak relational networks, their technology application is largely confined to short-term operational optimization. Furthermore, their credit expansion signals are prone to being misinterpreted as signs of financial distress, and their technology directives lack the support of embeddedness in social structures. As a result, the transformation from technology adoption to systemic resilience is difficult to achieve. This difficulty implies that growth-stage enterprises remain vulnerable points in the food system, where limited digital empowerment heightens the risk of supply chain disruptions and inventory shortfalls when external shocks occur.

## 6 Discussion: implications for food security

Food security ultimately depends on the capacity of food systems to sustain a continuous, affordable, and safe food supply in the face of recurring disruptions. Understanding the drivers of supply chain resilience is therefore of direct policy relevance. The empirical evidence presented in this study lends support to the view that firm-level digital transformation can translate into system-level resilience, with potential indirect implications for food security. We interpret our findings through the theoretical lenses that informed our study. From the perspectives of endogenous growth theory and induced technological change theory, digital innovation enhances system-level responsiveness and lowers coordination frictions.

Mediation analysis identifies two pathways through which digital innovation contributes to food security. The first is operational efficiency, reflected in current asset turnover and accounts receivable turnover. Faster inventory turnover reduces post-harvest losses and smooths seasonal supply fluctuations, while well managed receivables serve as an internal liquidity buffer that enables downstream firms to maintain purchases when credit conditions tighten. The second pathway concerns market expectations. Digital capabilities enhance market capitalization by improving investor assessments, which lowers the cost of capital for resilience investments and demonstrates reliability to trading partners, thereby facilitating long-term contractual relationships. Together, these mechanisms explain how digital innovation prevents localized liquidity stress from cascading into broader supply shortages. This dual pathway configuration provides a nuanced understanding of the micro level transmission from technological upgrading to food system stability, a channel that prior literature has largely left unexplored.

The enabling effect of digital innovation, however, is far from uniform. Heterogeneity analysis shows that the effect is significant in eastern regions, urban agglomerations, and non-old industrial bases, but insignificant in central and western regions, non-urban agglomeration areas, and old industrial bases. Likewise, significant effects are concentrated among non-state owned, large scale, and mature enterprises, with no such effects detected among state owned, small scale, or growth stage firms. These disparities are not merely statistical artifacts. Regions and firms that fail to benefit from digital empowerment are often those with weaker infrastructure and more constrained access to finance, conditions that make them inherently more susceptible to disruptions. The implication is that the very segments of the food system most vulnerable to shocks are precisely those least protected by digital innovation, potentially widening rather than narrowing resilience gaps.

It is important to emphasize that our empirical analysis directly examines agricultural supply chain resilience rather than food security per se. Our findings therefore speak to food security only indirectly—by identifying factors that enhance resilience, which in turn is widely recognized as a prerequisite for stable food systems. We caution against interpreting our results as direct causal evidence of improved food security outcomes.

This asymmetry acquires added significance from a global perspective. Chinese agricultural enterprises such as COFCO occupy strategic positions in the international food supply chain, controlling substantial shares of global grain procurement, shipping, and distribution. Any operational discontinuity in these nodes can propagate across borders, affecting global food prices and supply availability. Strengthening the resilience of Chinese agricultural firms through digital innovation therefore carries implications that extend well beyond domestic borders, contributing to the stability of global food systems by reinforcing the reliability of critical nodes through which a substantial portion of internationally traded agricultural commodities flows.

## 7 Conclusions, policy implications, and limitations

### 7.1 Conclusion

Using micro-level data on Chinese agricultural firms from 2010 to 2025, this study empirically investigates the impact of digital technology innovation on agricultural industry chain resilience, with a specific focus on its implications for food system stability. The analysis yields three key insights.

To begin with, digital technology innovation significantly strengthens agricultural industrial chain resilience, a result that remains robust across various specifications. In terms of stable food provisioning, this suggests that firm-level digital innovation lowers the probability of supply interruptions, thereby providing a micro-level technological route to ensuring continued food availability.

Turning to mechanisms, mediation analysis reveals that digital innovation enhances resilience through two channels. The first is operational efficiency, reflected in faster current asset turnover and accounts receivable turnover. Faster current asset turnover cuts inventory time and spoilage, while faster receivable turnover boosts liquidity and helps downstream partners maintain procurement, storage, and distribution under credit constraints. The second channel is market expectation. Digital innovation raises a firm’s market capitalization by improving investor assessments. A higher market capitalization lowers capital costs for resilience investments and signals credibility to supply chain partners, making long term contracts easier to sustain. Both channels together explain how digital innovation prevents localized disruptions from escalating into food shortages.

Regarding heterogeneity, the positive effect is concentrated in eastern regions, urban agglomerations, and non-old-industrial bases at the regional level, and in non-state-owned, large-scale, and mature enterprises at the firm level. Conversely, no significant effects are found in central and western regions, non-urban-agglomeration areas, old industrial bases, or in state-owned, small-scale, and growth-stage enterprises. These heterogeneous patterns carry critical implications for food system stability. Regions and firm types where digital technology innovation fails to enhance resilience constitute potential weak links in the food supply chain.

### 7.2 Policy implications

Based on the above findings, this study offers the following policy recommendations for safeguarding food security through digital technology innovation.

First, a technology incentive and diffusion system should be established across the entire agricultural chain. Policymakers should deploy R&D super deductions and digital transformation subsidies to lower-firms technology adoption costs. Public service platforms for technologies such as the Internet of Things and blockchain should be built to accelerate diffusion to small and medium sized entities. Refined governance rules for agricultural data factors are also needed to ensure that firm level digital gains translate into systemic resilience for food supply continuity.

Second, financial operations should be transformed from passive capital occupation to proactive efficiency-driven models. Leveraging the dual mediating pathways of current asset and accounts receivable turnover, firms should optimize asset allocation through precise demand forecasting, dynamic inventory management, and automated settlement. These practices reduce postharvest losses and smooth seasonal supply fluctuations. Enhanced digital risk control also accelerates debt recovery and capital reallocation, forging a positive cycle of efficiency improvement, capital release, and resilience reinforcement that directly supports stable food supply.

Third, a market-oriented financial strategy that channels investor sentiment into operational buffers should be developed. Drawing on the market expectation mechanism, agricultural firms can deploy digital solutions to enhance disclosure and demonstrate business sustainability, thereby lifting market valuation. A higher valuation brings down financing expenses for protective investments like temperature-controlled warehouses and alternative supply routes. It also conveys reliability to trading partners, facilitating enduring purchase agreements. This approach converts market confidence into fiscal cushions that stop isolated shocks from turning into chain wide food deficits.

Fourth, spatially and firm-level differentiated policies should be implemented to address the heterogeneous resilience deficits of vulnerable nodes in the food system. At the spatial level, cross-regional coordination mechanisms should be employed to facilitate factor mobility and information sharing, thereby preventing lagging regions from becoming structurally vulnerable nodes under systemic shocks. At the firm level, tailored measures are needed to strengthen the resilience of different types of market participants. The overarching objective of such differentiated interventions is to ensure the functional continuity and security of food supply chains in the face of external disruptions.

Fifth, Chinese agricultural enterprises should be repositioned as contributors to global food security rather than merely domestic suppliers. The sampled firms, notably COFCO, are both domestic industry anchors and pivotal nodes in the global supply chain. Policy support should extend beyond national boundaries, enabling these firms to participate in international governance and convert their resilience practices into global public goods. This would not only amplify China’s institutional voice but also cushion global market fluctuations by fortifying critical nodes, thus lending institutional weight to the maintenance of global food security.

Sixth, coordinated actions across governments, enterprises, and industry associations are essential to translating digital innovation into supply chain resilience. Governments should prioritize digital infrastructure in lagging regions and deploy targeted incentives to stimulate adoption. Agricultural enterprises should invest in digital capabilities and supply chain integration to capture resilience gains. Industry associations and financial institutions should facilitate knowledge diffusion and tailor financing instruments to lower adoption barriers. Collectively, these efforts address structural disparities, strengthen firm-level capacity, and ease financial constraints, forming a coherent pathway from digital adoption to supply chain resilience. This multi-stakeholder approach would enhance domestic agricultural stability and reinforce global food supply chains by fortifying critical nodes, thereby lending institutional weight to global food security.

Seventh, strengthening digital resilience across the agricultural chain requires a tiered strategy—short-term contingency, transitional consolidation, and long-term institutionalization. In the short term, a digital emergency revolving fund and forward-looking expectation indicators should be established. Smart logistics and early-warning systems validated during this phase should then be integrated into regional digital infrastructure, with priority given to addressing operational inefficiencies in central and western regions and among smaller-scale producers. Over the long term, resilience metrics should be integrated into the food security accountability framework, and agricultural data legislation should be enacted to institutionalize the lessons learned from earlier phases.

### 7.3 Limitations and future directions

Our measure captures firms’ publicly disclosed digital adoption rather than their unobservable innovation inputs or R&D expenditures. As such, it is best interpreted as an indicator of the extent of digital technology adoption reported in corporate disclosures, and should not be overinterpreted as a comprehensive proxy for all dimensions of digital innovation. Future research could complement our findings by incorporating alternative data sources, such as survey-based innovation metrics and firm-level R&D expenditure data, to capture a more holistic picture.

Beyond this measurement consideration, we acknowledge that our empirical strategy does not fully eliminate endogeneity concerns. Although we employ lagged explanatory variables to mitigate reverse causality, it remains plausible that more resilient firms are simultaneously better positioned to invest in digital innovation, raising the possibility of bidirectional relationships. We therefore frame our findings as evidence of a robust association rather than a definitive causal effect. Future studies could strengthen causal identification through instrumental variable approaches, quasi-natural experiments, or difference-in-differences designs that exploit exogenous shocks to digital adoption.

Compounding these empirical constraints, the generalizability of our results is inherently limited by the compositional nature of our sample, which is predominantly drawn from well-capitalized and formally structured agricultural enterprises in China. Our findings may not extend to the broader population of agricultural businesses, particularly smaller, family-run, or undercapitalized firms. We therefore encourage future research to extend our analysis to more diverse samples, including informal agricultural producers and micro-enterprises, to test the external validity of our conclusions and to explore potential heterogeneity across different types of agricultural entities.

## Data Availability

The original contributions presented in the study are included in the article/supplementary material, further inquiries can be directed to the corresponding authors.

## Footnotes

1 FAO. (n.d.). *What is food security?* Mesoamerica Hunger Free AMEXCID-FAO. Retrieved June 8, 2026, from https://www.fao.org/in-action/mesoamerica-sin-hambre/frequently-asked-questions/en/
